# Late Onset Takayasu Arteritis and Rheumatoid Arthritis

**DOI:** 10.1155/2012/523218

**Published:** 2012-07-15

**Authors:** K. E. Verweij, A. M. E. van Well, J. W. vd Sluijs, A. Dees

**Affiliations:** ^1^Department of Internal Medicine, Ikazia Hospital, Montessoriweg 1, 3083 AN Rotterdam, The Netherlands; ^2^Department of Surgery, Ikazia Hospital, Montessoriweg 1, 3083 AN Rotterdam, The Netherlands; ^3^Department of Radiology, Ikazia Hospital, Montessoriweg 1, 3083 AN Rotterdam, The Netherlands

## Abstract

We encountered the rare case of a 48-year-old Caucasian woman who developed Takayasu arteritis (TA) while suffering from seropositive rheumatoid arthritis (RA). Several studies have reported an association between TA and various autoimmune disorders, however, the concurrent presence of Takayasu arteritis and rheumatoid arthritis is described in only few cases in the literature to date. The exact nature of the relationship between TA and RA remains unknown. Perhaps the development of these two diseases represents non-specific systemic inflammatory changes in the presence of a hereditary background predisposing to both RA and TA.

## 1. Introduction

Takayasu arteritis (TA) is a rare, chronic vasculitis of unknown etiology that most frequently involves the aorta and its branches. It most frequently affects young Asian woman, with an age at onset usually between 10 and 40 years. In Europe the incidence is 1–3 per million per year. It has been suggested that the vasculitis may deteriorate via an autoimmune mechanism. Several studies have reported an association between TA and various autoimmune disorders such as systemic lupus erythematosus or systemic sclerosis. However, the concurrent presence of Takayasu arteritis and rheumatoid arthritis (RA) is described in only few cases in the literature to date.

We report the case of a 48-year-old Caucasian woman in whom Takayasu arteritis was diagnosed after treatment of seropositive rheumatoid arthritis for a year. 

## 2. Case Report

A 48-year-old Caucasian woman visited the outpatient clinic, complaining of pain and swelling in left knee and small joints in her hands. Laboratory findings showed a slightly elevation of erythrocyte sedimentation rate (ESR; 40 mm/h) and C-reactive protein (CRP; 28 mg/L). Rheumatoid factor (RF) level was <20 IU/mL, and anticyclic citrullinated peptide (anti-CCP) level was 94 IU/mL. Subsequent radiography showed bone atrophy around the wrist, suggesting RA. She was diagnosed as having seropositive rheumatoid arthritis. Treatment was started with prednisolone (15 mg/day) and methotrexate (15 mg/week), which improved her clinical symptoms and laboratory findings. Leflunomide (diseasemodifying antirheumatic drug (DMARD)) at 20 mg was additionally administered but was discontinued because of oral ulcerations. After a 6-month period, she remained stable on 7.5 mg prednisolone a day and 15 mg methotrexate a week.

However, a year later, she presented with fatigue and pain in both upper extremities. She did not experience weight loss or low-grade fever. The RA remained stable with the aforementioned therapy. Upon physical examination, a blood pressure difference between the left and the right arm was noted, respectively, 109/71 mmHg and 93/71 mmHg. Furthermore, a murmur was noticed over the left carotid artery. Pulsations of both radial arteries were reduced. Laboratory tests showed a slight leukocytosis (15 × 10^9^/L), CRP 14 mg/L, and ESR 14 mm/h. 

**Figure 1:**
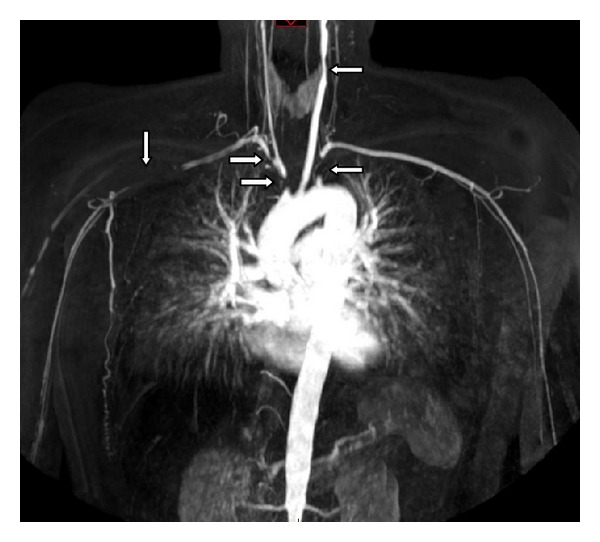
MRA with extensive vascular stenotic lesions.

Subsequent MRA and angiography demonstrated extensive vascular stenotic lesions (Figure 1). In the right truncus brachiocephalicus, an occlusion at its origin from the aortic arch was found. The right subclavian artery showed two important constrictions with a significant stenotic lesion of the proximal branch and distal a complete occlusion. The left subclavian artery was constricted in its proximal part. There was also occlusion of the left common carotid artery at its origin as well in its distal part. Furthermore, both left common iliac artery and right external iliac artery showed stenotic lesions. Positron emission tomography (PET) scan demonstrated increased fluorodeoxyglucose (FDG) uptake along both brachial arteries, suggestive of active vasculitis. Despite the late onset, the diagnosis TA was made according to the Sharma modified diagnostic criteria, approximately a year after the patient was diagnosed with RA. We started therapy with a higher-dose prednisolone (40 mg/day). Despite our treatment, the patient's condition deteriorated with intermittent claudication of both legs. Subsequently Bosentan (Tracleer, 250 mg/day) was added, and she underwent a percutaneous transluminal angioplasty (PTA) with placement of a stent across the stenotic segment of the left common iliac artery and right external iliac artery. After placement of the stents, her clinical symptoms were markedly relieved. Subclavian stenting has been scheduled in due time. Due to the development of hypotension of 77/74 mmHg on Bosentan therapy, the dose was adjusted. Lately she has been stable on 125 mg a day. Antitumor necrosis factor (TNF)-*α*  therapy as a next step in the treatment will be considered.

## 3. Discussion

Takayasu arteritis is a rare chronic inflammatory disease of unknown aetiology that results in stenosis or occlusion or the large vessels. It is typically characterized by granulomatous inflammation of the aorta and its major branches. The most frequently affected vessel is the subclavian (left greater than right) followed by common carotid (left greater than right), brachiocephalic and vertebral arteries [1, 2].

The first description of TA was published in 1830 by Yamamoto describing the case of a 45-year-old man with persistent fever who developed absent pulses in upper extremities associated with dyspnoea [3]. However, the disease is named after Mikito Takayasu, an ophthalmologist who gave the first scientific presentation of TA in 1905. 

Takayasu arteritis typically affects young females from East Asia with a median age at diagnosis of 28 years, very rarely occurring after age 40 [1, 2]. 

The diagnosis may be difficult due to initial nonspecific symptoms. Nonspecific features include fever, night sweats, malaise, weight loss, arthralgia, myalgia, and mild anaemia. As the inflammation progresses and vascular lesions develop, symptoms related to ischaemia become apparent [2]. Diseases to consider in the differential diagnosis of Takayasu arteritis include other causes of vasculitis: syphilis, tuberculosis, systemic lupus erythematosus (SLE), Behçet's disease, Bechterew disease, Buerger disease (thromboangiitis obliterans), and Kawasaki disease. Most of these diseases have other specific features that enable the diagnosis. 

Over the years various sets of diagnostic criteria have been developed to assist in the diagnosis of TA. The most recent is the Sharma modified diagnostic criteria who were developed in 1995 [4] (Table 1). These criteria consist of three major and ten minor factors. The presence of two major criteria, or one major and two minor criteria, or four minor criteria indicates a high probability of Takayasu's arteritis.

Here we present a patient who was diagnosed with TA based on the Sharma modified diagnostic criteria. This case is remarkable on the one hand because of the advanced age at diagnosis of TA in combination with her Caucasian gender. On the other hand, the case is exceptional because of the concurrent presence of RA. We wondered whether there might be a relationship between the two diseases and secondly if there is an explanation for the late onset of TA. 

The specific pathogenesis of TA remains to be clarified however, it has been suggested that the vasculitis may deteriorate via an autoimmune mechanism with infiltration of cytotoxic T lymphocytes and the release of proinflammatory cytokines such as tumor necrosis factor (TNF)-*α*  [5]. The fact that associations with various autoimmune disorders have been described, such as SLE or systemic sclerosis, supports this theory [6, 7]. 

However, despite a similar pathogenesis with a prominent role of TNF-*α*, the association between RA and TA is rare [8]. In the literature to date only few cases of the concurrent presence of TA and RA have been described. Some of these reports suggest that the age at diagnosis of TA in patients with RA is higher in comparison to patients with isolated TA [9, 10]. In a series of 20 patients with RA, the mean age at diagnosis of TA was more advanced than the typical age at diagnosis of isolated TA (resp., 55 years versus 28 years) [9]. A possible explanation is that the nonspecific initial symptoms of TA, which are also known as symptoms of RA, were regarded as RA-related symptoms.

Our patient was treated with immunosuppressive agents. However, under this therapy, she developed multiple vascular stenotic lesions in progressive claudication of both legs. This course is typically for patients with TA since it is a systemic vascular disease which can progress and ultimately cause vital organ ischaemia. 

Early treatment with glucocorticoids and cytotoxic agents is important to avoid rapid progression of the vascular lesions. However, relapses are frequent, and 25% of the patients develop progressive disease despite conventional treatment. Antitumor necrosis factor (TNF)-*α*  therapy may be a useful treatment in patients with refractory TA [11]. Especially in our patient, it may be effective because of a similar pathogenesis with the role of TNF-*α*  in both RA and TA [5, 8]. Furthermore, balloon angioplasty and stenting are important in the management of TA. In patients with TA aortitis, 85% are improved or cured by endovascular intervention. One should consider early endovascular intervention for symptomatic stenoses [1]. An alternative is surgical intervention; however, surgery is not recommended in an area of active disease due to a higher incidence of anastomotic stricture or aneurysm [1]. 

In this patient we started with a new therapy for TA, Bosentan, an endothelin receptor blocker. The mechanism is thought to involve the inhibition of vasoconstriction, cell hyperplasia, and hypertrophy and therefore to improve the blood flow. It also inhibits joint swelling and protects against join inflammation and destruction in patients with RA. 

In summary, we report the case of a Caucasian woman who was diagnosed with late-onset TA (age 48) after being treated for RA for a year. As mentioned above, the exact nature of the relationship between TA and RA remains unknown. Perhaps the development of these two diseases represents non-specific systemic inflammatory changes in the presence of a hereditary background predisposing to both RA and TA. We emphasize the importance of considering TA when ischemic symptoms occur during the course of RA.

## Figures and Tables

**Table 1:** Modified Sharma diagnostic criteria for Takayasu arteritis [1].

Major criteria	Minor criteria
(1) Left midsubclavian artery stenosis or occlusion (2) Right midsubclavian artery stenosis or occlusion (3) Signs and symptoms (>1 month duration) (i) Limb claudication (ii) Absent pulses or >10 mmHg blood pressure differential in arms (iii) Exercise ischaemia (iv) Neck pain (v) Fever (vi) Amaurosis fugax (vii) Syncope (viii) Dyspnoea (ix) Palpitations (x) Blurred vision	(1) ESR > 20 mm/hr (2) Carotid bruit (3) Hypertension >140/90 mmHg (4) Aortic regurgitation (5) Pulmonary artery stenosis or aneurysm (6) Left midcommon carotid artery stenosis or occlusion (7) Distal third innominate artery stenosis or occlusion (8) Descending thoracic aortic stenosis, occlusion, or irregularity (9) Abdominal aortic narrowing, aneurysm, or irregularity (10) Angiographic evidence of coronary artery disease in a patient <30 years old without atherosclerotic risk factors

Two major, or one major and two minor, or four minor criteria indicate a high probability of Takayasu arteritis. ESR: erythrocyte sedimentation rate.
